# Apolipoprotein A-I enhances insulin-dependent and insulin-independent glucose uptake by skeletal muscle

**DOI:** 10.1038/s41598-018-38014-3

**Published:** 2019-02-04

**Authors:** Shudi Tang, Fatiha Tabet, Blake J. Cochran, Luisa F. Cuesta Torres, Ben J. Wu, Philip J. Barter, Kerry-Anne Rye

**Affiliations:** School of Medical Sciences, Faculty of Medicine, UNSW Sydney, Australia

## Abstract

Therapeutic interventions that increase plasma high density lipoprotein (HDL) and apolipoprotein (apo) A-I levels have been reported to reduce plasma glucose levels and attenuate insulin resistance. The present study asks if this is a direct effect of increased glucose uptake by skeletal muscle. Incubation of primary human skeletal muscle cells (HSKMCs) with apoA-I increased insulin-dependent and insulin–independent glucose uptake in a time- and concentration-dependent manner. The increased glucose uptake was accompanied by enhanced phosphorylation of the insulin receptor (IR), insulin receptor substrate-1 (IRS-1), the serine/threonine kinase Akt and Akt substrate of 160 kDa (AS160). Cell surface levels of the glucose transporter type 4, GLUT4, were also increased. The apoA-I-mediated increase in glucose uptake by HSKMCs was dependent on phosphatidylinositol-4,5-bisphosphate 3-kinase (PI3K)/Akt, the ATP binding cassette transporter A1 (ABCA1) and scavenger receptor class B type I (SR-B1). Taken together, these results establish that apoA-I increases glucose disposal in skeletal muscle by activating the IR/IRS-1/PI3K/Akt/AS160 signal transduction pathway. The findings suggest that therapeutic agents that increase apoA-I levels may improve glycemic control in people with type 2 diabetes.

## Introduction

Apolipoprotein (apo) A-I is the most abundant apolipoprotein constituent of high density lipoproteins (HDLs)^1^. In normal human plasma 5–10% of the total apoA-I exists in a lipid-free or lipid-poor form that accepts cholesterol from peripheral cells expressing the ATP-binding cassette transporter A1 (ABCA1)^2^. Lipid-free/lipid-poor apoA-I also interacts with scavenger receptor class B type 1 (SR-B1) to activate signal transduction pathways^3^.

HDLs and apoA-I have anti-diabetic properties^4^. We, and others, have reported that apoA-I increases insulin secretion from pancreatic beta-cells in an ABCA1-dependent manner. HDLs also increase insulin secretion from pancreatic beta-cells, but in an ABCG1-dependent manner^5^. Furthermore, mice that are deficient in apoA-I have impaired glucose tolerance^6^, while overexpression of apoA-I increases insulin sensitivity^7^. Similar findings have been reported in people with type 2 diabetes, where infusions of reconstituted HDL consisting of apoA-I complexed with phosphatidylcholine increase plasma insulin levels and reduce plasma glucose levels^8^. Torcetrapib, a small molecule inhibitor of cholesteryl ester transfer protein activity that increases plasma HDL cholesterol and apoA-I levels by approximately 70%, also improves glycaemic control in people with type 2 diabetes^9^. There is evidence that HDLs and apoA-I may mediate these effects by increasing glucose disposal in skeletal muscle via activation of the AMP-activated protein kinase (AMPK) signalling pathway^6,8^. We have also reported that a single infusion of apoA-I increases glucose disposal and phosphorylation in skeletal muscle in *db/db* mice, both in the presence and in the absence of insulin^10^.

Insulin-dependent glucose uptake into skeletal muscle is initiated by the binding of insulin to the α-subunit of the insulin receptor (IR), and phosphorylation of tyrosine residues in the IR β-subunit (IRβ)^11^. This leads to phosphorylation of tyrosine residues in insulin receptor substrate-1 (IRS-1)^12^, and the p85 subunit of phosphatidylinositol 3-kinase (PI3K)^13^. Phosphorylated PI3K then activates a downstream signal transduction pathway that phosphorylates serine/threonine kinase Akt (protein kinase B)^14,15^ and the Rab GTPase-activating protein, Akt substrate of 160 kDa (AS160)^16^. This culminates in the translocation of glucose transporter 4 (GLUT4) to the cell surface, and increases glucose uptake^17^. The present study establishes that apoA-I enhances this signalling pathway and the translocation of GLUT4 to the cell surface.

## Results

### ApoA-I increases glucose uptake in HSKMCs

**Figure 1:**
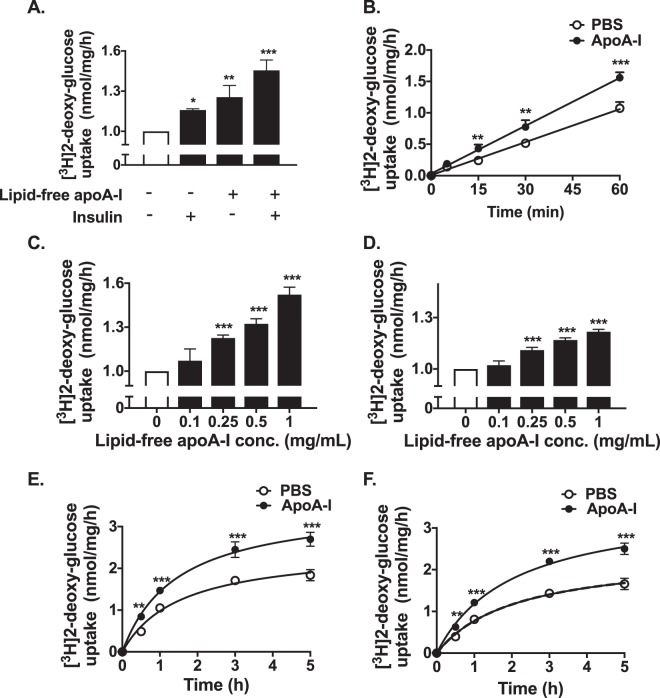
ApoA-I increases glucose uptake into HSKMCs. (**A**) HSKMCs were incubated at 37 °C for 16 h in serum-free MEM-α with or without apoA-I (1 mg/mL final concentration). The apoA-I was removed and the cells were incubated at 37 °C for 1 h with or without insulin (0.1 µmol/L final concentration). (**B**) Glucose uptake was assessed from 5 to 60 min in HSKMCs pre-incubated with (closed symbols) or without (open symbols) apoA-I as in Panel A, then incubated for a further 1 h in the presence of insulin (0.1 µmol/L final concentration). (**C**,**D**) HSKMCs were incubated at 37 °C for 16 h in serum-free MEM-α with or without apoA-I (0.1–1 mg/mL final concentration). The apoA-I was removed, and the cells were incubated for 1 h in the (**C**) presence or (**D**) absence of insulin (0.1 µmol/L final concentration). (**E**,**F**) Glucose uptake was assessed from 0.5 to 5 h in HSKMCs pre-incubated with (closed symbols) or without (open symbols) apoA-I as in Panel A, then incubated for a further 1 h in the (**E**) presence or (**F**) absence of insulin (0.1 µmol/L final concentration). Glucose uptake was determined as described in Materials and Methods. Values represent the mean ± SD of three independent experiments. Glucose uptake was normalized to 1 nmol/mg/h for cells incubated under basal conditions (open bar, Panels A, C,D). *p < 0.05, **p < 0.01, ***p < 0.001 vs. control (open bars and circles).

As reported previously, incubation of human skeletal muscle cells (HSKMCs) with insulin alone increased glucose uptake from 1.0 nmol/mg/h (Fig. 1A, open bar) to 1.16 ± 0.01 nmol/mg/h (Fig. 1A, closed bar) (*p* < 0.05)^6^. Incubation of HSKMCs with lipid-free apoA-I alone increased glucose uptake to 1.26 ± 0.09 nmol/mg/h (*p* < 0.01 vs. incubation with insulin alone), while incubation with apoA-I plus insulin increased glucose uptake to 1.46 ± 0.08 nmol/mg/h (*p* < 0.001 vs. incubation with insulin alone) (Fig. 1A, closed bar). Evidence that uptake of glucose in the absence (open circles) and presence (closed circles) of apoA-I was linear during the 1 h incubation period is shown in Fig. 1B. ApoA-I also increased insulin- dependent and –independent glucose uptake under basal and high glucose conditions (Supplemental Fig. I).

The ability of lipid-free apoA-I to enhance insulin-dependent and -independent glucose uptake was concentration-dependent. Incubation of HSKMCs with apoA-I at a concentration of 0.25 mg/mL increased insulin-dependent (Fig. 1C) and insulin–independent (Fig. 1D) glucose uptake from 1.0 nmol/mg/h (Fig. 1C and D, open bar) to 1.23 ± 0.02 nmol/mg/h (*p* < 0.01) and 1.11 ± 0.02 nmol/mg/h (*p* < 0.001), respectively, (Fig. 1C and D closed bars). At an apoA-I concentration of 1 mg/mL, insulin-dependent and insulin-independent glucose uptake was 1.52 ± 0.05 nmol/mg/h and 1.22 ± 0.01 nmol/mg/h, respectively (*p* < 0.001 for both) (Fig. 1C and D closed bars).

ApoA-I also increased glucose uptake into HSKMCs in a time-dependent manner. When the cells were incubated with PBS in the absence of apoA-I, insulin-dependent glucose uptake into HSKMCs was 0.49 ± 0.01 nmol/mg/h at 0.5 h and 1.84 ± 0.13 nmol/mg/h at 5 h (Fig. 1E, open circles). In the presence of apoA-I insulin-dependent glucose uptake increased to 0.84 ± 0.04 nmol/mg/h at 0.5 h and 2.7 ± 0.17 nmol/mg/h at 5 h (Fig. 1E, closed circles) (*p* < 0.001 vs. incubation without apoA-I). Insulin-independent glucose uptake into HSKMCs was 0.40 ± 0.01 nmol/mg/h at 0.5 h and 1.66 ± 0.14 nmol/mg/h at 5 h (Fig. 1F, open circles). In the presence of apoA-I insulin-independent glucose uptake was 0.63 ± 0.02 nmol/mg/h at 0.5 h and 2.5 ± 0.14 nmol/mg/h at 5 h (Fig. 1F, closed circles) (*p* < 0.001 vs. incubation with PBS).

### Lipid-free apoA-I activates the IR/IRS-1/PI3K/Akt/AS160 signalling pathway in HSKMCs

**Figure 2:**
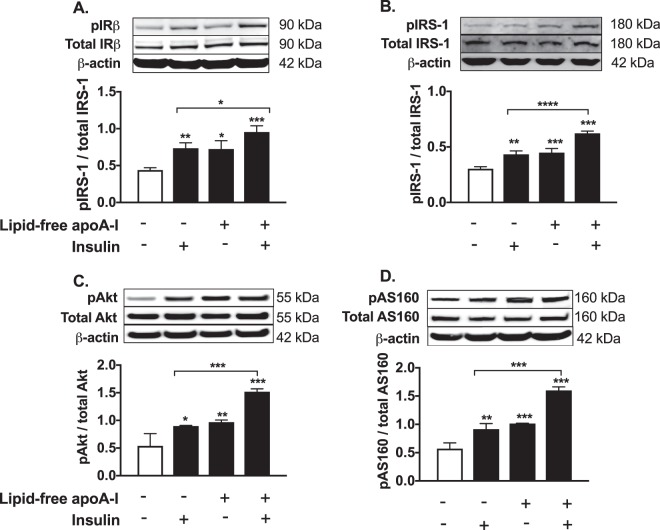
ApoA-I phosphorylates IRβ, IRS1, Akt and AS160 in HSKMCs. HSKMCs were pre-incubated at 37 °C for 16 h in serum-free MEM-α with or without lipid-free apoA-I then incubated with insulin as described in the legend to Fig. 1. The cells were lysed, subjected to SDS-PAGE and immunoblotted for (**A**) total and phosphorylated IRβ, (**B**) total and phosphorylated IRS-1, (**C**) total and phosphorylated Akt, (**D**) total and phosphorylated AS160 using β-actin as the loading control. Values represent the mean ± SD of three independent experiments.*p < 0.05, **p < 0.01, ***p < 0.001 vs. control (open bars).

To determine if apoA-I increased glucose uptake by activating the IR/IRS-1/PI3K/Akt/AS160 signal transduction pathway, phosphorylation of IRβ, IRS-1, Akt, and AS160 was assessed by western blotting. Incubation with insulin alone or apoA-I alone increased the HSKMC phosphorylated IRβ (pIRβ)/total IRβ ratio from a control value of 0.44 ± 0.03 (Fig. 2A, open bar) to 0.73 ± 0.07 (*p* < 0.01) and 0.72 ± 0.11 (*p* < 0.05), respectively (Fig. 2A, closed bars). Incubation with apoA-I plus insulin increased the pIRβ/total IRβ ratio to 0.95 ± 0.08 (Fig. 2A, closed bars) (*p* < 0.001 vs. control; *p* < 0.05 vs. cells treated insulin alone). Incubation with apoA-I plus insulin increased the pIRS-1/total IRS-1 ratio to 0.62 ± 0.02 (Fig. 2B, closed bars) (*p* < 0.001 vs. control; *p* < 0.0001 vs. cells treated with insulin alone). The phosphorylated Akt (pAkt)/total Akt ratio in control HSKMCs was 0.54 ± 0.22 (Fig. 2C, open bar), compared to 0.9 ± 0.01 for cells incubated with insulin alone (Fig. 2C, closed bar) (*p* < 0.05) and 0.97 ± 0.04 for cells incubated with apoA-I (*p* < 0.01). Incubation of HSKMCs with insulin plus apoA-I increased the pAkt/total Akt ratio to 1.52 ± 0.05 (*p* < 0.001 compared to control cells and cells treated with insulin alone). The phosphorylated AS160 (pAS160)/total AS160 ratio, which was 0.57 ± 0.1 in the control HSKMCs (Fig. 2D, open bar), increased to 0.91 ± 0.1 (*p* < 0.01) and 1.01 ± 0.006 (*p* < 0.001), respectively, when the cells were incubated with insulin or apoA-I (Fig. 2D, closed bars), and to 1.6 ± 0.06 when the cells were incubated with apoA-I plus insulin (*p* < 0.001 compared to control and cells treated with insulin alone).

Taken together, these results indicate that apoA-I activates the IR/IRS-1/PI3K/Akt/AS160 insulin signaling pathway in an insulin-dependent and an insulin–independent manner.

### ApoA-I increases insulin-stimulated glucose uptake into HSKMCs in an IRS-1 and PI3K-dependent manner

**Figure 3:**
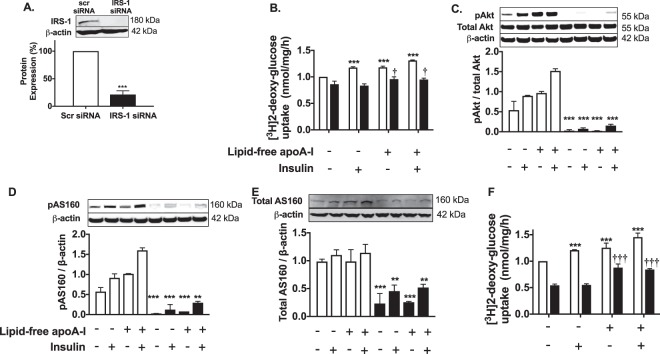
ApoA-I increases insulin-dependent glucose uptake in HSKMCS in an IRS-1 and PI3K/Akt/AS160-dependent manner. (**A**) HSKMCs were transfected with scrambled siRNA (scr siRNA) or IRS-1 siRNA. IRS-1 protein expression was quantified by immunoblotting. (**B**) Scr-siRNA- (open bars) and IRS-1 siRNA-transfected (closed bars) HSKMCs were pre-incubated with or without apoA-I then incubated in the presence or absence of insulin. Glucose uptake was quantified as described in the legend to Fig. 1. (**C–E**) Total and phosphorylated Akt, and AS160 were determined by immunoblotting after incubation in the presence (closed bars) and absence (open bars) of wortmannin. (**F**) Glucose uptake in HSKMCs was determined in the presence (closed bars) and absence (open bars) of wortmannin as described in Materials and Methods. All values represent the mean ± SD of three independent experiments. Glucose uptake was normalized to 1 nmol/mg/h for control scr siRNA-transfected cells. ***p < 0.001 vs. control for scr siRNA-transfected cells or cells incubated in the absence of wortmannin; ^†^p < 0.05 versus control IRS-1 siRNA-transfected cells; ^†††^p < 0.001 versus cells incubated with wortmannin under control conditions.

The effect of lipid-free apoA-I on the IRS-1/PI3K/Akt/AS160 insulin signaling pathway was further investigated by transfecting HSKMCs with IRS-1 siRNA. Transfection of HSKMCs with IRS-1 siRNA decreased IRS-1 protein expression by 79 ± 7% (Fig. 3A) (p < 0.001 vs. cells transfected with scrambled (scr) siRNA).

Incubation of the scrambled siRNA-transfected HSKMCs with insulin only or apoA-I only increased glucose uptake from a normalized value of 1 nmol/mg/h to 1.17 ± 0.02 and 1.18 ± 0.02 nmol/mg/h, respectively (Fig. 3B, open bars) (*p* < 0.001 for both). When the scrambled siRNA-transfected cells were incubated with apoA-I plus insulin, glucose uptake further increased to 1.31 ± 0.01 nmol/mg/h (*p* < 0.001 vs. control). Glucose uptake decreased to 0.86 ± 0.06 nmol/mg/h when HSKMCs were transfected with IRS-1 siRNA and incubated in the absence or presence of insulin (Fig. 3B, closed bars). Incubation of the IRS-1 siRNA-transfected HSKMCs with apoA-I alone or apoA-I plus insulin increased glucose uptake modestly to 0.96 ± 0.04 and 0.96 ± 0.03 nmol/mg/h, respectively (*p* < 0.05 for both compared to control cells transfected with IRS-1 siRNA).

To determine if activation of PI3K/Akt was required for the apoA-I-mediated uptake of glucose into HSKMCs, incubations were also carried out with the PI3K/Akt inhibitor, wortmannin. Akt (Fig. 3C), and AS160 phosphorylation (Fig. 3D) as well as total AS160 levels (Fig. 3E) were all decreased when HSKMCs were incubated with wortmannin (Fig. 3C–E, closed bars), irrespective of whether or not apoA-I and insulin were present. Incubation of HSKMCs with insulin or apoA-I in the absence of wortmannin increased glucose uptake from 1.0 nmol/mg/h to 1.21 ± 0.01 nmol/mg/h and 1.25 ± 0.08 nmol/mg/h, respectively (Fig. 3F, open bars) (*p* < 0.001 for both). Incubation with apoA-I plus insulin in the absence of wortmannin further increased glucose uptake to 1.45 ± 0.07 nmol/mg/h (*p* < 0.001 vs. control).

Glucose uptake was decreased in the wortmannin-treated HSKMCs, irrespective of whether or not insulin was present in the incubation (Fig. 3F, closed bars). Incubation of wortmannin-treated HSKMCs with apoA-I increased glucose uptake (Fig. 3F, closed bars, p < 0.001), but the previously observed synergistic effect of insulin plus apoA-I on glucose uptake was abolished. Overall, these results indicate that apoA-I increases insulin-dependent glucose uptake in HSKMCs via IRS-1- and PI3K/Akt-dependent and independent pathways.

### ApoA-I increases GLUT4 translocation to the plasma membrane in HSKMCs

**Figure 4:**
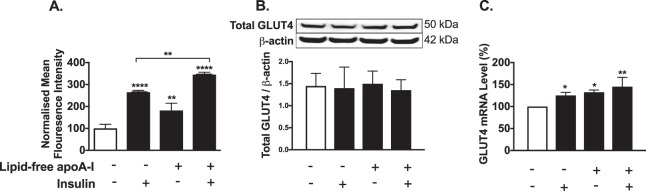
ApoA-I increases GLUT4 translocation to the HSKMC plasma membrane. HSKMCs were pre-incubated at 37 °C for 16 h with or without apoA-I then incubated in the presence and absence of insulin as described in the legend to Fig. 1. (**A**) Cell surface GLUT4 was labeled with an antibody that specifically recognizes the extracellular domain of the protein and analysed by flow cytometry. (**B**) Total cell lysates were immunoblotted for GLUT4 using β-actin as a loading control. **(C)** GLUT4 mRNA levels were quantified by real-time PCR. Values represent the mean ± SD of three independent experiments. *p < 0.05, **p < 0.01, ****p < 0.0001 vs. control (open bar).

The normalised plasma membrane GLUT4 level was 100 ± 18.7 mean fluorescence intensity units (MFI) in untreated HSKMCs (Fig. 4A, open bar). Inclusion of insulin or apoA-I in the incubation increased the plasma membrane GLUT4 level to 264.7 ± 8.547 (*p* < 0.0001 vs. control) and 181.8 ± 33 MFI, respectively (*p* < 0.01 vs. control) (Fig. 4A, closed bars). Incubation with insulin plus apoA-I further increased the HSKMC plasma membrane GLUT4 level to 345.1 ± 10.92 MFI (*p* < 0.001 vs. control; *p* < 0.01 vs. cells incubated with insulin alone). Incubation of HSKMCs with insulin or apoA-I did not affect total cell GLUT4 levels (Fig. 4B), but increased GLUT4 mRNA levels by 25.2 ± 7.0% and 32.3 ± 5.3%, respectively (Fig. 4C, *p* < 0.05 for both). Glycosylation of GLUT4 can cause heterogeneous bands in western blots. However, GLUT4 western blots can also be well focussed, as is the case in Fig. 4B. This variation is dependent on the amount of protein loaded onto the gel, whether or not a stacking gel was used and the duration of electrophoresis. High sample loading, use of a stacking gel and a low running voltage all have the capacity to produce poorly focused GLUT4 bands in western blots^18–21^. Incubation of HSKMCs with apoA-I plus insulin increased cellular GLUT4 mRNA levels by 45.3 ± 21% (*p* < 0.01 vs. control). Incubation of HSKMCs with apoA-I did not alter IR, IRS-1, PI3K and Akt mRNA levels (Supplemental Fig. II A–D).

### ABCA1 and SR-B1 regulate apoA-I-mediated glucose uptake in HSKMCs

**Figure 5:**
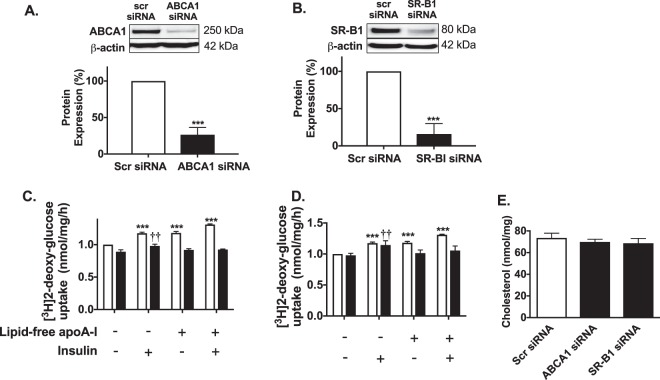
ApoA-I increases glucose uptake in HSKMCs in an ABCA1- and SR-B1-dependent manner. HSKMCs were transfected with scrambled siRNA (scr siRNA), ABCA1 siRNA or SR-B1 siRNA. The cells were lysed and ABCA1 (**A**) and SR-B1 (**B**) protein levels were quantified by immunoblotting. Glucose uptake was measured in the ABCA1 siRNA-transfected (**C**) and SR-B1 siRNA-transfected cells (**D**) (closed bars) as described in the legend to Fig. 1. The cholesterol content of HSKMCs transfected with ABCA1 siRNA and SR-B1 siRNA was quantified as described in Materials and Methods (**E**). Values represent the mean ± SD of three independent experiments. Glucose uptake was normalized to 1 nmol/mg/h for control, scr siRNA-transfected cells (open bars). ***p < 0.001 vs. cells transfected with control scr-siRNA; ^††^p < 0.01 vs. cells transfected with ABCA1 siRNA and SR-B1 siRNA then incubated under control conditions.

Transient transfection of HSKMCs with ABCA1 siRNA and SR-B1 siRNA decreased ABCA1 and SR-B1 protein expression by 74 ± 10% (Fig. 5A) and 84 ± 14%, respectively (Fig. 5B, *p* < 0.001 for both). Addition of insulin, apoA-I or insulin plus apoA-I to scrambled siRNA-transfected HSKMCs increased glucose uptake from 1 nmol/mg/h to 1.17 ± 0.02, 1.18 ± 0.02 and 1.31 ± 0.01 nmol/mg/h, respectively (Fig. 5C,D, open bars) (*p* < 0.001 for all). Glucose uptake by HSKMCs that were transfected with ABCA1 siRNA and incubated with PBS was 0.89 ± 0.03 nmol/mg/h (Fig. 5C, closed bar). Incubation of the ABCA1 siRNA-transfected cells with insulin increased glucose uptake to 0.98 ± 0.03 nmol/mg/h (*p* < 0.01 versus control). Inclusion of apoA-I in the incubations of ABCA1 siRNA-transfected HSKMC did not increase glucose uptake, irrespective of whether or not insulin was present (Fig. 5C, closed bars). Incubation with insulin increased glucose uptake into the SR-B1 siRNA-transfected HSKMCs from 0.98 ± 0.03 to 1.15 ± 0.06 nmol/mg/h (Fig. 5D, closed bars) (*p* < 0.05). ApoA-I, by contrast, did not increase glucose uptake into the SR-B1-transfected HSKMCs in either the presence or absence of insulin (Fig. 5D, closed bars). Incubation of non-transfected HSKMCs with apoA-I did not alter ABCA1 and SR-B1 mRNA levels (Supplemental Fig. II E,F).

To determine if the effects of ABCA1 and SR-B1 knockdown on glucose uptake could be explained by an increase in HSKMC cholesterol content, cholesterol levels in the transfected cells were quantified by HPLC. The cholesterol content of the scrambled siRNA-transfected cells was 73.6 ± 4.4 nmol/mg protein (Fig. 5E, open bar), compared to 69.9 ± 2.5 nmol/mg protein and 68.6 ± 4.5 nmol/mg protein for the ABCA1 siRNA- and SR-B1 siRNA-transfected cells, respectively (Fig. 5E, closed bars). This indicates that ABCA1 and SR-B1 regulate apoA-I-mediated glucose uptake by HSKMCs by a mechanism that is independent of their role in maintaining cellular cholesterol homeostasis.

## Discussion

The present study establishes that apoA-I, the predominant HDL apolipoprotein, significantly increases insulin-dependent and insulin–independent glucose disposal in skeletal muscle by a mechanism that involves phosphorylation of IRβ and IRS-1, activation of the PI3K/Akt/AS160 signal transduction pathway and increased translocation of GLUT4 to the cell surface. The ability of insulin to activate the IRβ/IRS-1/PI3K/Akt/AS160 pathway and regulate GLUT4 translocation to the cell membrane is well known^12,22^, but the capacity of apoA-I to enhance these events directly has not been reported previously. The outcome of the present study also provides a possible explanation for earlier reports showing that interventions that increase HDL levels, such as reconstituted HDL infusions and inhibition of cholesteryl ester transfer protein activity, improves glycaemic control and insulin sensitivity in people with type 2 diabetes^8,23^.

One of the most interesting observations to emerge from the present study is that apoA-I can act alone, as well as synergistically with insulin, to increase IR, IRS-1, Akt and AS160 phosphorylation. This is consistent with previous reports showing that apoA-I increases glucose uptake into skeletal muscle cells in the absence of insulin, and that apoA-I and insulin have a synergistic effect on AMPK-dependent glucose uptake in C2C12 myotubes^6,8^. It is also consistent with our previous report showing that apoA-I increases insulin-dependent glucose uptake in skeletal muscle from diabetic *db/db* mice by increasing insulin sensitivity in the muscle^10^. Previous studies have shown that apoA-I knockout mice have reduced glucose tolerance^6^, whereas overexpression of apoA-I in transgenic mice increases insulin sensitivity^7^. The results in the present study indicate that apoA-I may mediate these effects directly by increasing glucose disposal in skeletal muscle, although the possible involvement of an apoA-I-mediated improvement in pancreatic beta cell function, as we have reported previously^5,24^, cannot be excluded.

We also found that knockdown of IRS-1 and inhibition of Akt and AS160 phosphorylation with wortmannin attenuated apoA-I-mediated insulin-dependent glucose uptake, but did not affect the ability of apoA-I to promote insulin-independent glucose uptake in HSKMCs (Fig. 3). This may reflect the differential regulation of insulin- and AMPK-induced glucose transport in skeletal muscle cells, with reports showing that AS160 phosphorylation is mediated both directly and indirectly by insulin and AMPK^22,25,26^. It does not, however, explain the synergistic phosphorylation of IRS-1 and Akt that occurred when HSKMCs were incubated with apoA-I plus insulin. Hence, in addition to the AMPK pathway, this observation raises the possibility that apoA-I may enhance glucose uptake by additional, yet-to-be identified insulin-independent pathways.

The current study also establishes that apoA-I increases GLUT4 translocation to the cell surface in the presence and absence of insulin. This is consistent with a previous report showing that HDL can increase GLUT4 translocation to the adipocyte surface^27^. As activation of IRS-1/PI3K/Akt/AS160 insulin signal transduction has been reported to promote GLUT4 translocation to the cell surface in skeletal muscle^28^, it is likely that activation of this pathway by apoA-I was responsible for the increase in cell membrane GLUT4 content in Fig. 4. As apoA-I also activates the AMPK pathway in skeletal muscle^6,8^, this pathway may have further contributed to the observed increase in GLUT4 translocation^29^.

The absence of change in total GLUT4 protein levels in HKSMCs that were incubated with apoA-I was unexpected. This is possibly because GLUT4 is continuously recycling between the cell surface and intracellular compartments^30^, with the increase in cell surface GLUT4 levels reflecting either increased GLUT4 translocation from intracellular compartments to the cell membrane, decreased recycling of cell surface GLUT4 back to intracellular compartments, or an increase in GLUT4 synthesis. It is also important to note that although insulin primarily promotes GLUT4 translocation to the cell surface, GLUT4 continues to recycle and undergo degradation in insulin-stimulated cells^31^. This may explain why GLUT4 mRNA levels were increased by apoA-I, but GLUT4 protein expression in the cells was unaltered.

The present results also establish that the ability of apoA-I to increase insulin-dependent and insulin–independent glucose uptake by skeletal muscle is regulated by ABCA1 and SR-B1. This is in agreement with a previous report showing that ABCA1 is involved in apoA-I-mediated, insulin-independent glucose uptake in skeletal muscle cells^8^. The present study extends this finding by establishing that the ability of apoA-I to increase insulin-dependent and insulin-independent glucose uptake by skeletal muscle also requires SR-B1. This is of particular interest as SR-B1 is known to activate several signal transduction pathways^3^. The result showing that ABCA1 and SR-B1 knockdown did not affect cellular cholesterol content indicates that regulation of apoA-I-mediated glucose uptake by ABCA1 and SR-B1 is independent of apoA-I acting as an acceptor of cellular cholesterol.

In conclusion, this study provides compelling evidence that apoA-I increases glucose disposal in skeletal muscle, thus supporting a role for HDL in reducing insulin resistance and improving glycaemic control in people with type 2 diabetes. This study further strengthens recent observations showing that HDL have anti-diabetic properties. It also suggests that HDL-raising interventions may be beneficial in the management of people with pre-diabetes as well as individuals with type 2 diabetes.

## Methods

### Isolation of apoA-I

HDLs were isolated from pooled samples of autologously donated human plasma (Healthscope Pathology, Adelaide, South Australia, AUS) by sequential ultracentrifugation in the 1.063< d <1.21 g/mL density range and delipidated as described^32^. ApoA-I was isolated from the resulting apoHDL by chromatography on a Q-Sepharose Fast-Flow column^33^. The apoA-I preparations appeared as a single band following electrophoresis on SDS-polyacrylamide PhastGels (GE Healthcare Bio-Sciences, San Francisco, CA, USA) and Coomassie Blue staining.

### Cell culture

Primary HSKMCs (#150–05 f, Cell Applications, San Diego, CA, USA) were cultured at 37 °C in 5% CO_2_ (v/v) in Minimum Essential Medium-α (MEM-α; #12571063, Gibco, Life Technologies, Carlsbad, CA, USA) containing 10% (v/v) FBS. When 90% confluent, the cells were plated into 12-well plates (3 × 10^4^ cells/mL) and cultured in MEM-α containing 2% (v/v) horse serum for 4–5 days. Differentiation into myotubes was monitored by phase contrast microscopy.

### Glucose uptake by HSKMCs

Fully differentiated HSKMCs were incubated at 37 °C for 16 h in serum-free MEM-α in the presence or absence of lipid-free apoA-I (1 mg/mL final concentration). The apoA-I was removed and the cells were incubated in fresh MEM-α for 1 h with or without insulin (0.1 µmol/L final concentration; #ART0103A, ARC, St Louis, MO, USA). For determination of [^3^H]2-deoxy-glucose uptake the cells were washed with *Krebs-Ringer Bicarbonate (*KRB) buffer containing 2 mmol/L sodium pyruvate (pH7.4). Glucose uptake was initiated by incubating the cells for 0.5–5 h with 10 µmol/L [^3^H]2-deoxy-glucose (37 kBq/mL final concentration) in KRB buffer in the presence or absence of insulin (0.1 µmol/L final concentration, #I9278, Sigma, St Louis, MO, USA). The cells were then rinsed with ice-cold Dulbecco’s PBS, lysed with SDS (0.05%, w/v) and subjected to liquid scintillation counting. Non-carrier-mediated glucose uptake was determined by incubating the cells for 15 min with cytochalasin B (10 µmol/L, #C2743, Sigma) prior to [^3^H]2-deoxy-glucose uptake^34^. The results were corrected for cell protein and non-carrier-mediated glucose uptake. The values for the control cells that were incubated in the absence of insulin and apoA-I were normalised to 1 nmol/mg protein/h. All experiments were carried out in triplicate. The results are representative of three or more independent experiments.

For incubation with wortmannin, fully differentiated HSKMCs were incubated for 1 h in serum-free MEM-α with wortmannin (0.1 µmol/L final concentration, #W3144, Sigma), then incubated for 16 h with wortmannin in the presence or absence of apoA-I (1 mg/mL final concentration). The apoA-I was removed, and the cells were incubated for 1 h with wortmannin with or without insulin (0.1 µmol/L final concentration). [^3^H]2-deoxy-glucose uptake was determined as described above.

### IRS-1, ABCA1 and SR-B1 knockdown

Near-confluent HSKMCs (1.7 × 10^4^ cells/mL) were plated in 12-well collagen-coated plates. Skeletal muscle differentiation medium was added to the cells the following day. After 3 days (~48 h prior to full differentiation), the cells were transfected for 16 h with scrambled siRNA, IRS-1 siRNA, ABCA1 siRNA or SR-B1 siRNA (0.1 µmol/mL final concentration for all; ON-TARGET SMART pool siRNA, GE Healthcare, San Francisco, CA, USA) using DharmaFECT 4 siRNA transfection reagent (#T-2004–03, GE Healthcare).

### GLUT4 translocation

GLUT4 translocation was measured as described previously^35^. Briefly, HSKMCs were incubated at 37 °C for 16 h with or without apoA-I (1 mg/mL). Primary anti-GLUT4 (#sc-1608, Santa Cruz Biotechnology, Dallas, TX, USA) and secondary DyLight488 donkey anti-goat (#ab96931, Abcam, Cambridge, MA, UK) antibodies were combined and maintained at room temperature for 10 min. The cells were washed with PBS and incubated for 30 min with the antibody complex in the presence and absence of insulin (0.1 μmol/L final concentration). After washing and fixing the cells with 2.5% (v/v) neutral buffered formalin for 20 min, GLUT4 translocation to the cell surface was analyzed using a FACSverse flow cytometer (BD Biosciences, San Jose, CA, USA).

### Western blotting

Fully differentiated HSKMCs were incubated at 37 °C for 16 h in serum-free MEM-α with or without apoA-I (1 mg/mL final concentration). The apoA-I was removed, and the cells were incubated with or without insulin (0.1 µmol/L final concentration) for 30 min. The cells were then washed with cold PBS and lysed with 50 mmol/L Na pyrophosphate, 50 mmol/L NaF, 50 mmol/L NaCl, 5 mmol/L EDTA-Na_2_, 5 mmol/L EGTA-Na_2_, 2 mmol/L Na_3_VO_4_, 10 mmol/L HEPES (pH 7.4), 0.1% Triton X-100 (v/v), 1 mmol/L PMSF, and a protease inhibitor cocktail (1:1000 (v/v)). The cell lysates (30 µg protein) were subjected to SDS-PAGE, transferred electrophoretically to a nitrocellulose membrane, and incubated with primary antibodies at dilutions of 1:3,000 (v/v) for anti-β-actin (#A1978, Sigma), and 1:1,000 (v/v) for all other antibodies. The membrane was then incubated with horseradish peroxidase-conjugated secondary antibodies (Santa Cruz, CA, USA). Antibodies against phospho-IRβ (tyr1345) (#3026), IRβ (#3020), phospho-Akt (ser473) (#4058), Akt (pan) (#4685), IRS-1 (#2390), phospho-AS160 (ser588) (#8730), and AS160 (#2670) were from Cell Signalling (Beverly, MA, USA). The phospho-IRS1 antibody (Tyr612, #09–431) was from Merck Millipore (Billerica, MA, USA). The ABCA1 antibody (#NB400–105) was from Novus Biologicals (Littleton, Colorado, USA), and the SR-B1 antibody (#1971–1) was from Abcam (Cambridge, UK). The blots were developed with ECL and quantified using Image Lab Analysis Software (Bio-Rad Laboratories Inc., CA, USA).

### Real-time PCR

Total RNA was isolated from HSKMCs using an RNeasy Mini Kit (Qiagen, Venlo, Netherlands) and reverse transcribed (100 ng RNA) using an iScript cDNA Synthesis Kit (Bio-Rad). Real-time PCR was performed with the MyiQ single colour real-time PCR detection system (Bio-Rad) using the iQ SYBR Green Supermix (Bio-Rad) in a BioRad iQ5 thermocycler. Relative changes in mRNA levels were determined by the ΔΔCT method. Results are presented as the ratio of the gene of interest to β-actin. Primer sequences are shown in Supplemental Table I.

### HSKMC cholesterol content

HSKMCs were lysed in water (500 µL). The lysates (400 µL) were added to water (600 µL) containing EDTA-Na_2_ (200 mmol/L, 10 µL) and BHT (0.2 mmol/L, 10 µL). Methanol:hexane (7.5 mL, 1:2 (v/v)) was added to the samples, which were mixed and centrifuged at 2,000 *g* for 5 min. The hexane layer was collected and dried, dissolved in isopropanol/acetonitrile (70/30 (v/v), 200 µL) and applied to a Supelco ODS LC-18 column (25 × 0.46 cm, 5 µm particle size) with a 2-cm Pelliguard column (Sigma) attached to a Shimadzu HPLC system (Kyoto, Japan). Cholesterol levels were quantified at 205 nm with reference to cholesterol standards (Sigma)^36^.

### Statistical analysis

Results are presented as mean ± SD of at least three independent experiments. Multiple comparisons were analysed by one-way ANOVA with Tukey’s post-test, or two-way ANOVA with Bonferroni post-test (Prism 5, Graphpad Software, La Jolla, CA). A value of *p* < 0.05 was considered significant.

## Supplementary information

Supplementary Information
