# Supplementary material for: Apolipoprotein A-I enhances insulin-dependent and insulin-independent glucose uptake by skeletal muscle
Source: Sci Rep. 2019 Feb 4;9:1350. doi: 10.1038/s41598-018-38014-3 (PMC6362284; doi:10.1038/s41598-018-38014-3)

SUPPLEMENTAL INFORMATION

APOLIPOPROTEIN A-I ENHANCES INSULIN-DEPENDENT AND INSULIN-INDEPENDENT GLUCOSE UPTAKE BY SKELETAL MUSCLE

by

Shudi Tang[a], Fatiha Tabet[a], Blake J. Cochran[a], Luisa F. Cuesta Torres[a], Ben J. Wu[a], Philip J. Barter[a], and Kerry-Anne Rye[a*]

[a]School of Medical Sciences, Faculty of Medicine, UNSW Sydney

*Correspondence to Professor Kerry-Anne Rye, Level 4 East, Wallace Wurth Building, Faculty of Medicine, School of Medical Science, UNSW Sydney, Sydney, Australia, NSW, 2052

Tel: +61 2 9385 1219; Fax: +61 2 9385 1797.

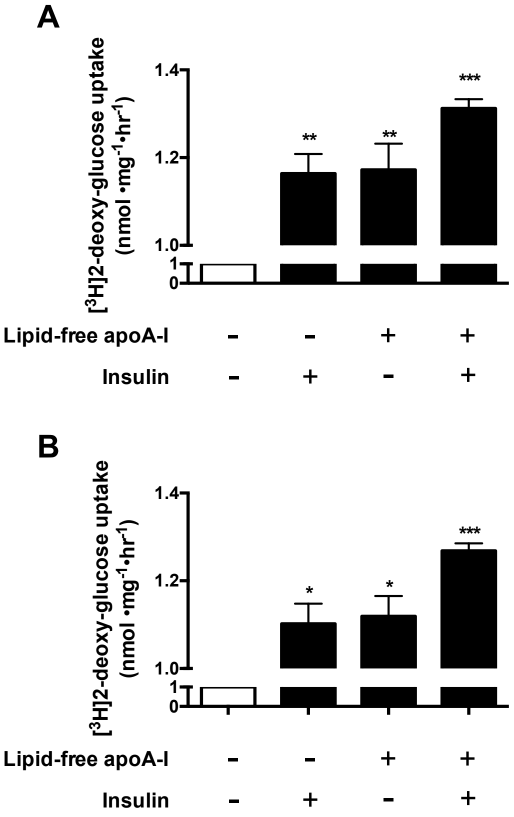

Supplemental Figure I. ApoA-I increases glucose uptake into HSKMCs in an insulin-dependent and -independent manner under basal and high glucose conditions. HSKMCs were incubated at 37 °C for 16 h in serum-free MEM-α under **(A)** low (2.8 mmol) glucose conditions and **(B)** high (25 mmol) glucose conditions with or without apoA-I (1 mg/mL final concentration). The apoA-I was removed and the cells were incubated at 37 °C for 1 h with or without insulin (0.1 μmol/L final concentration). Glucose uptake was determined as described in Materials and Methods. Values represent the mean±SD of three independent experiments. Glucose uptake was normalized to 1 nmol/mg/h for cells incubated under basal conditions (open bar).

*p<0.05, **p<0.01, ***p<0.001 vs. incubation under basal conditions.

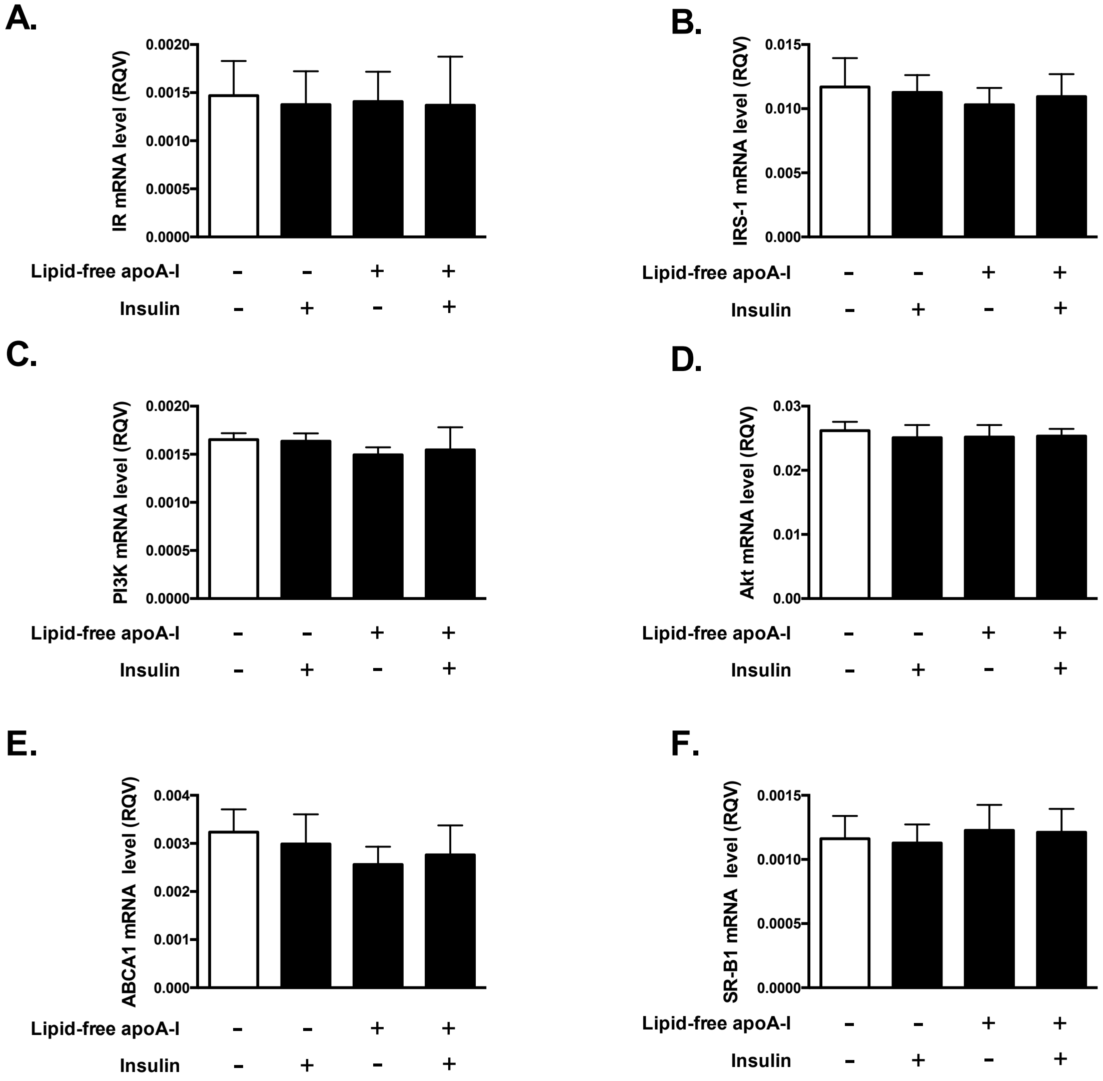

Supplemental Figure II. ApoA-I does not alter IR, IRS1, PI3K, Akt, ABCA1 or SR-B1 mRNA levels. HSKMCs were incubated at 37 °C for 16 h in serum-free MEM-α with or without apoA-I (1 mg/mL final concentration). The apoA-I was removed and the cells were incubated at 37 °C for 1 h with or without insulin (0.1 μmol/L final concentration). mRNA levels of **(A)** IR, **(B)** IRS-1, **(C)** PI3K, **(D)** Akt, **(E)** ABCA1 and **(F)** SR-B1 were measured by real time PCR. Values represent the mean±SD of three independent experiments.

Supplemental Table I: Sequence of oligonucleotide primers.

**Target Gene**	**Primer Sequence**
IR	**F:** 5’-CTGTAGTCCCAGTGCTAATC-3’
	**R:** 5’-ATAAAATCGGCATTCCCTGT-3’
IRS-1	**F:** 5’-ACTTGAGCTACGGTGACGTG-3’
	**R:** 5’-GCGGAACTCATCACTCATGG-3’
Akt	**F:** 5’-TGAACGAGTTTGAGTACCTG-3’
	**R:** 5’-GGATCTTCATGGCGTAGTAG-3’
PI3K	**F:** 5’-TGACGCTTTCAAACGCTATC-3’
	**R:** 5’-CAGAGAGTACTCTTGCATTC-3’
	**R:** 5’-ACCCCAATGTTGTACCCAAACT-3’
ABCA1	**F:** 5’-GAGCGGGTCAGCAAAGCAA-3’
	**R:** 5’-GTCCAATGCCTGCGACAGAT-3’
SR-B1	**F:** 5’-GCACACTCCTTGTTCCTGG-3’
	**R:** 5’-GTCCAATGCCTGCGACAGAT-3’
GLUT4	**F:** 5’-GCACACTCCTTGTTCCTGG-3’
	**R:** 5’-GTCCAATGCCTGCGACAGAT-3’
β-actin	**F:** 5’-CGGGAAATCGTGCGTGACAT-3’
	**R:** 5’-AAGGAAGGCTGGAAGAGTGC-3’

Full blot Fig. 2A.

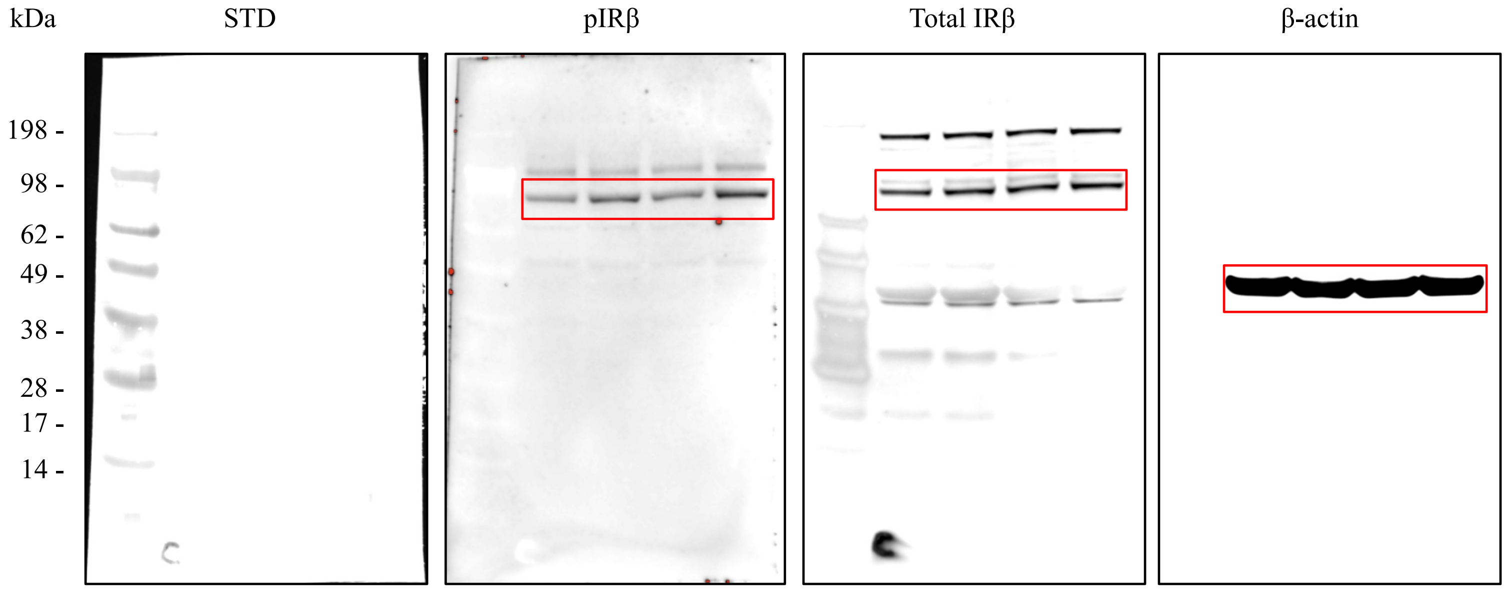

Full blot Fig. 2B.

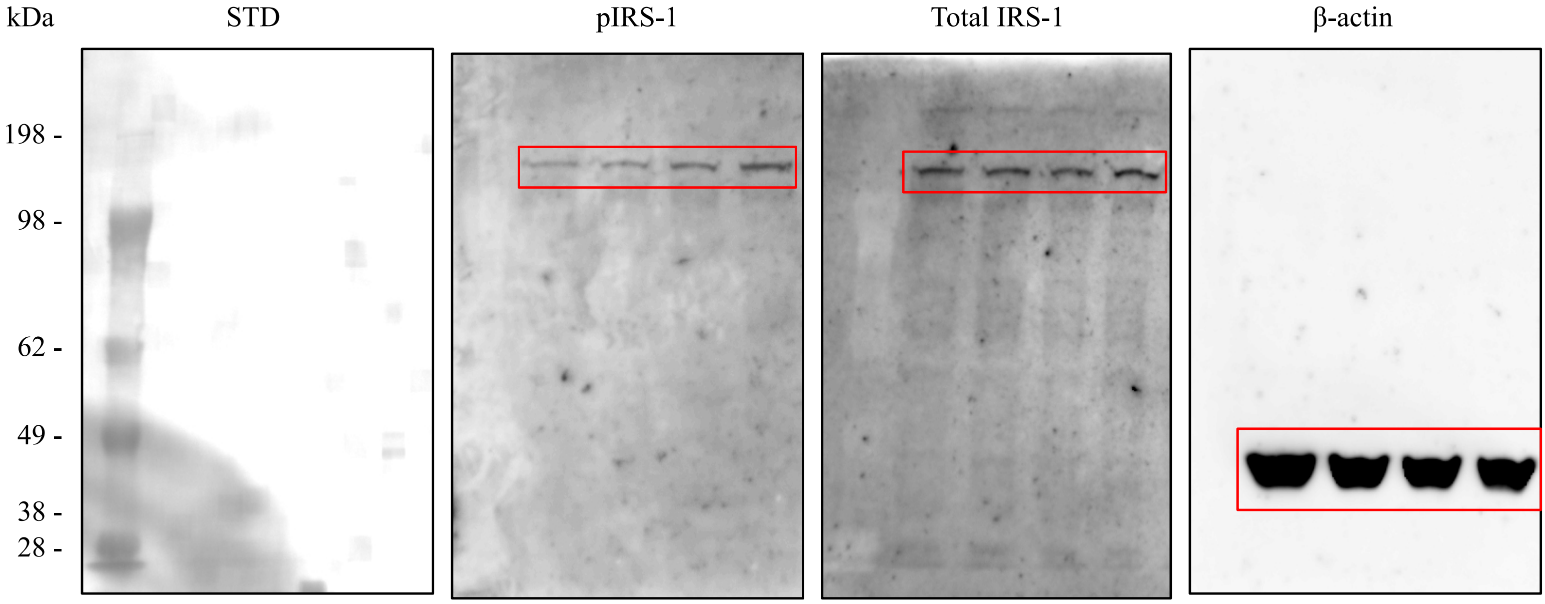

Full blot Fig. 2C.

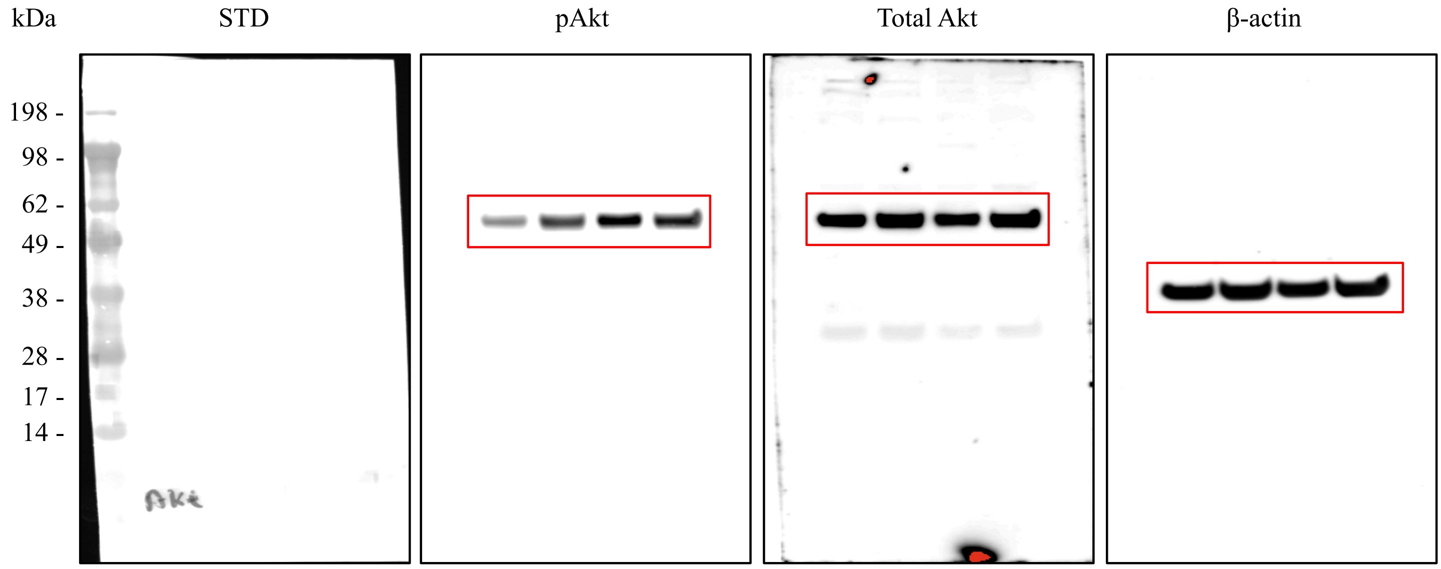

Full blot Fig. 2D.

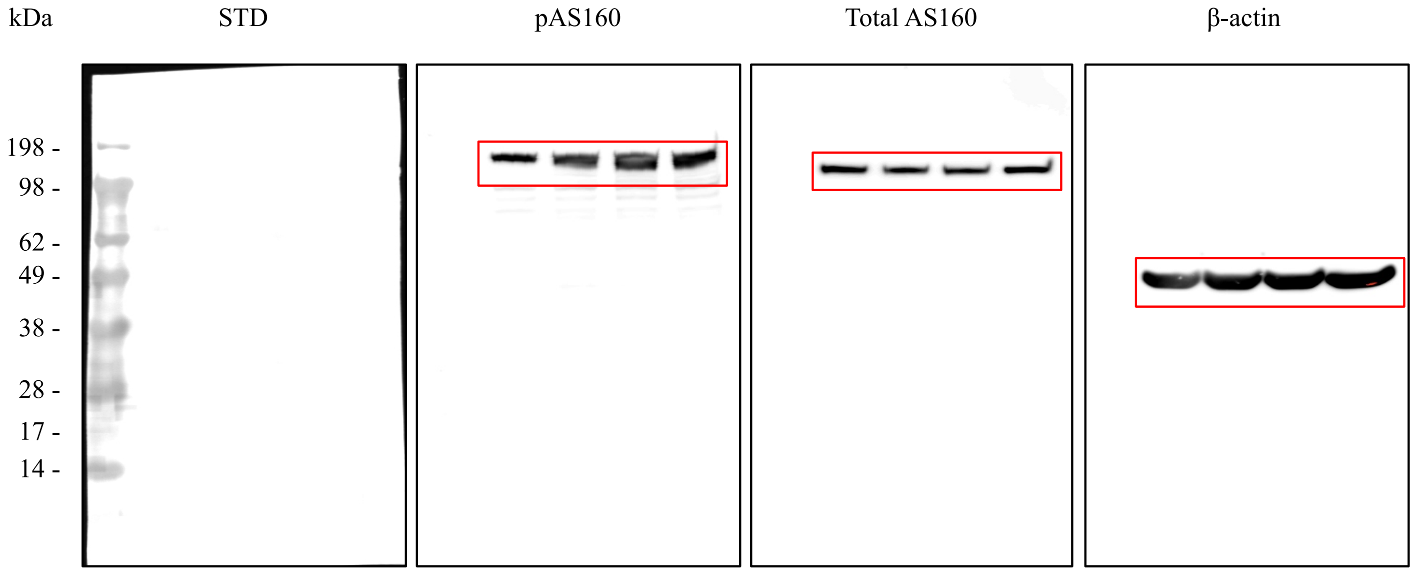

Full blot Fig. 3A.

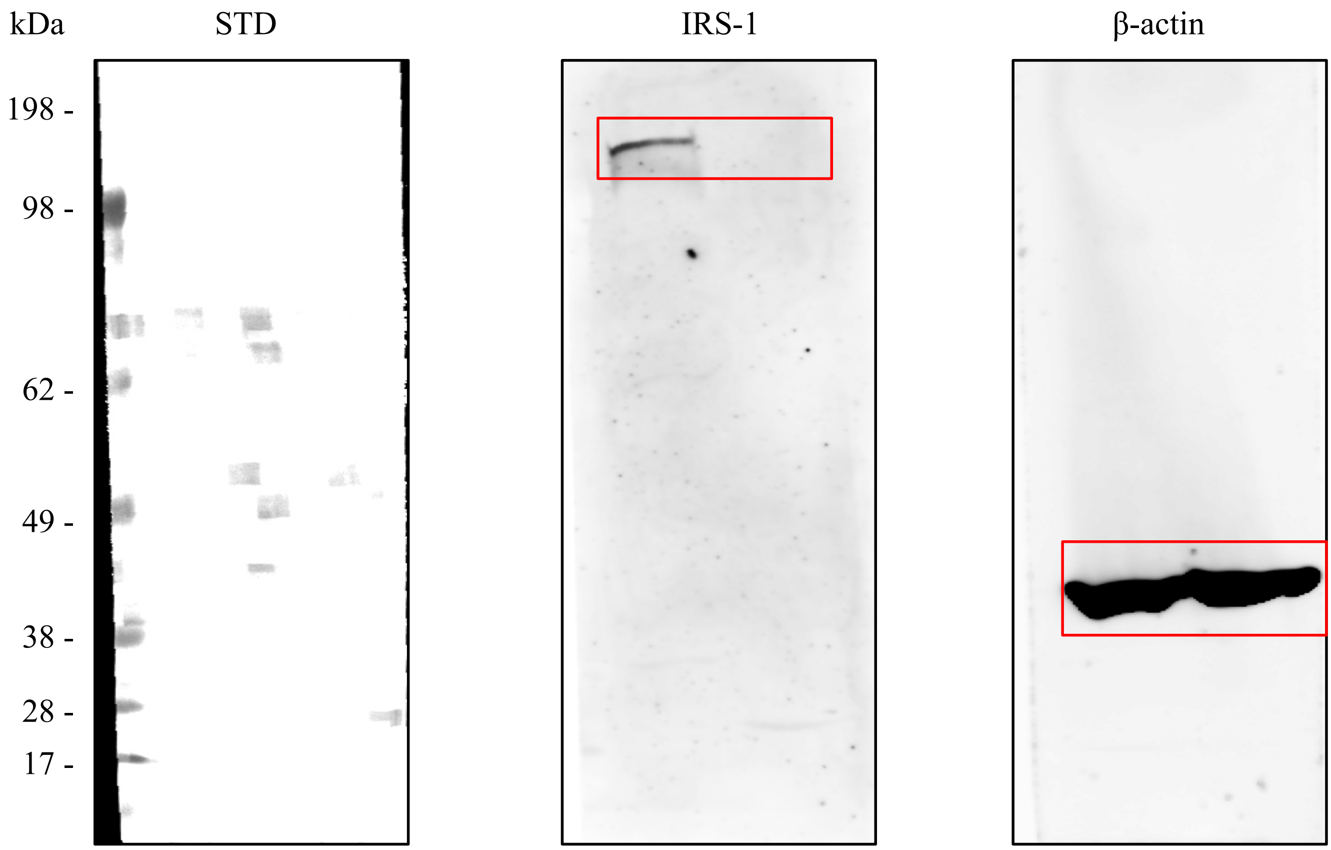

Full blot Fig. 3C.

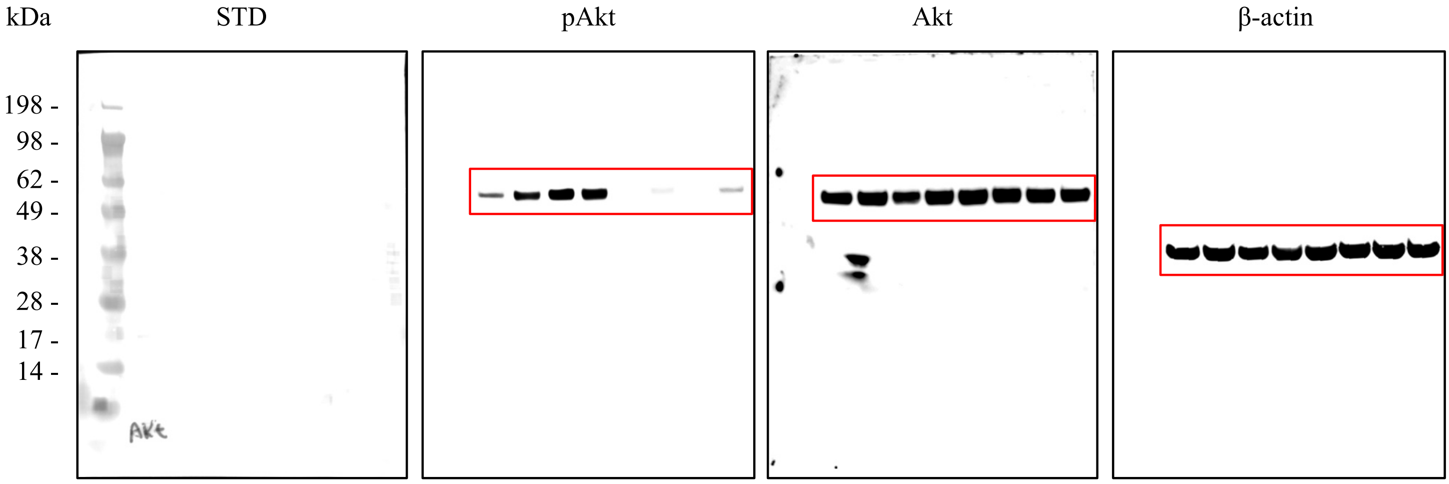

Full blot Fig. 3D.

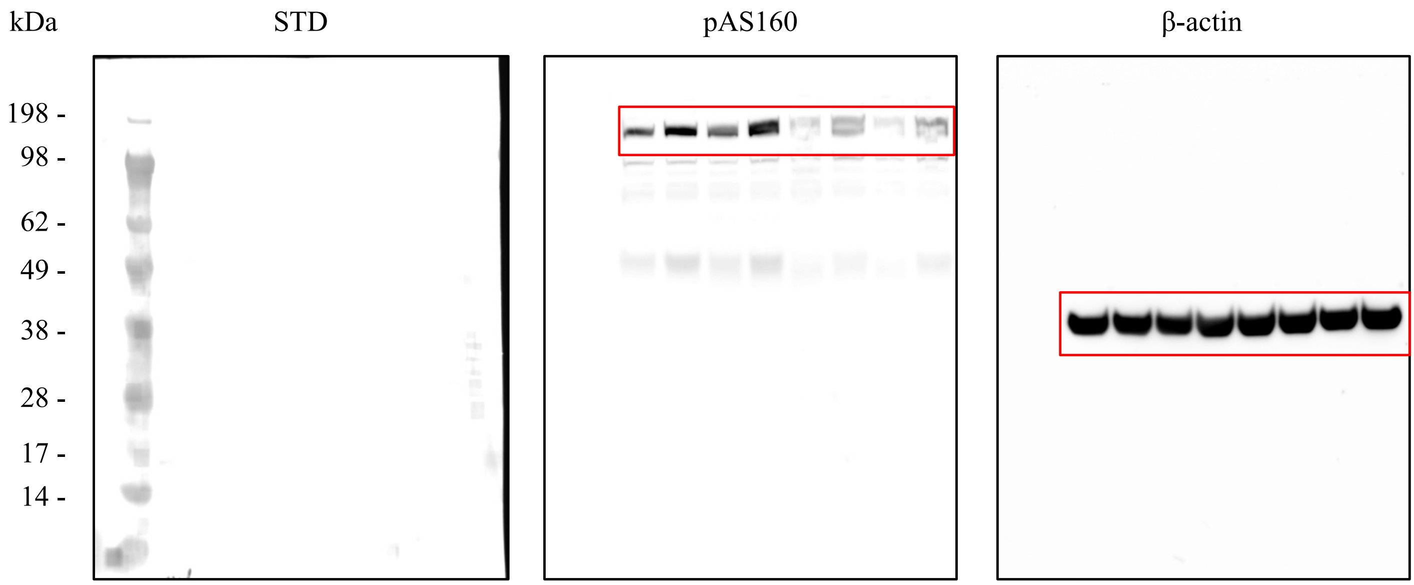

Full blot Fig. 3E.

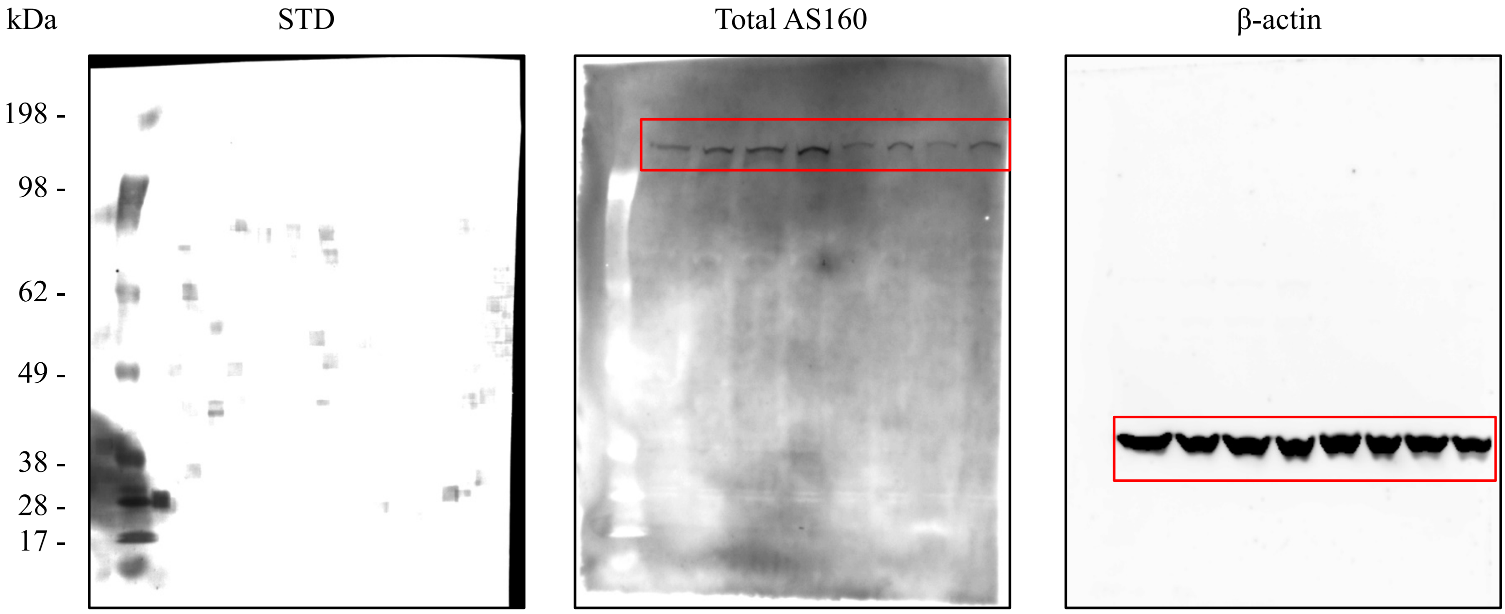

Full blot Fig. 4B

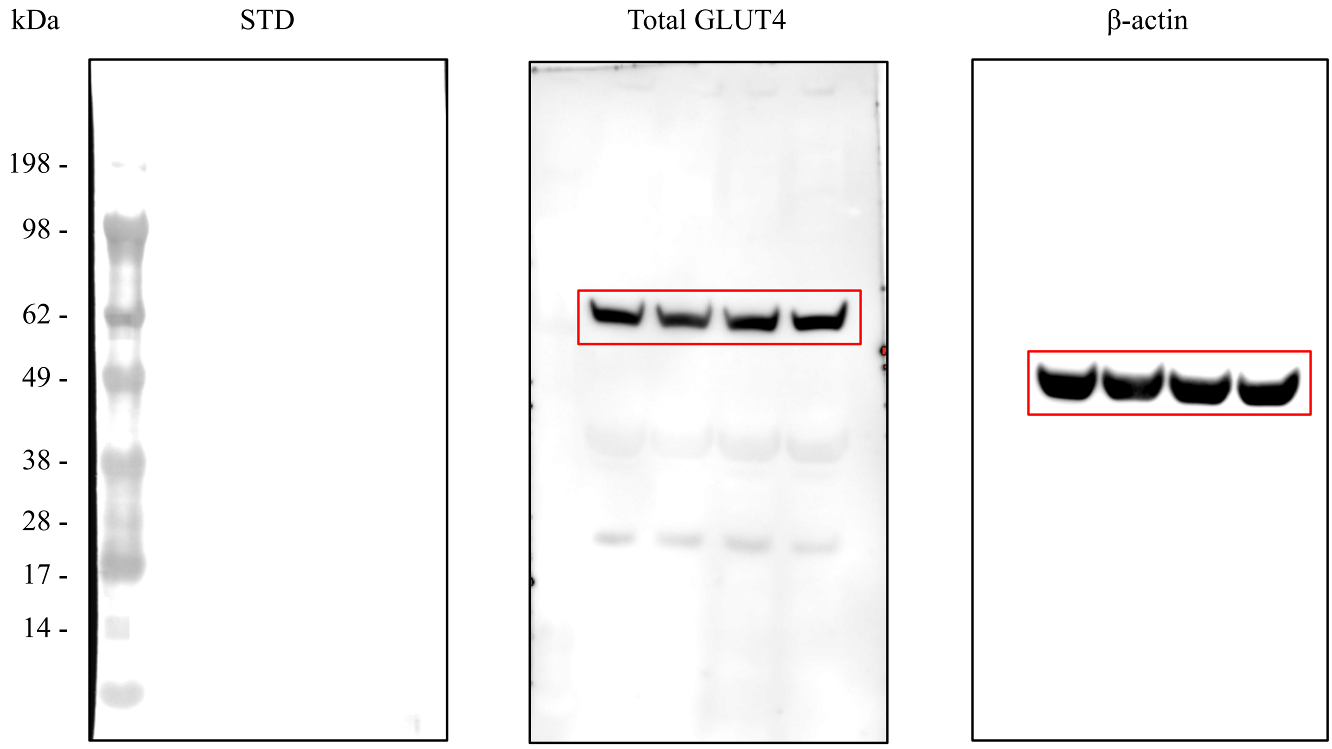

Full blot Fig. 5A.

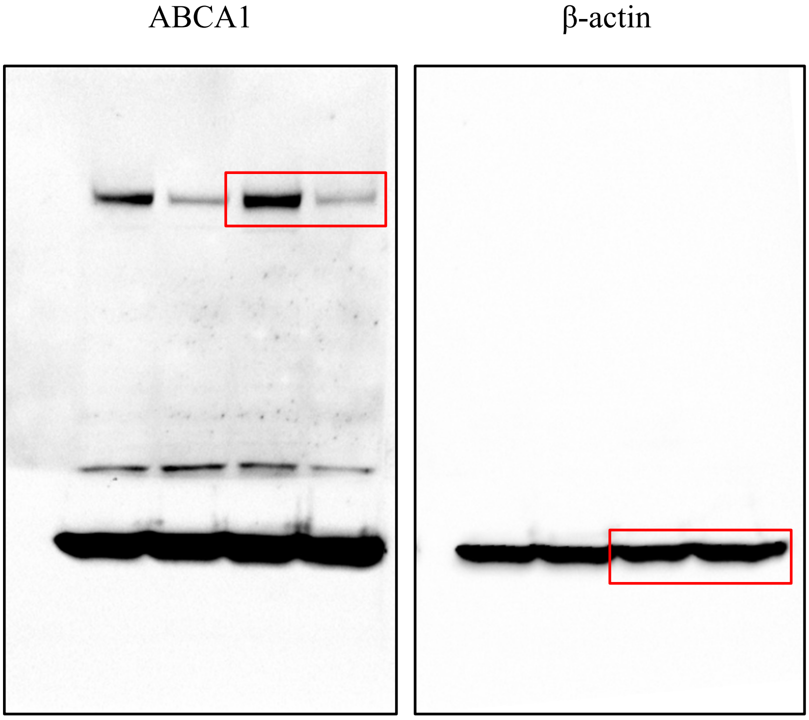

Full blot Fig. 5B.

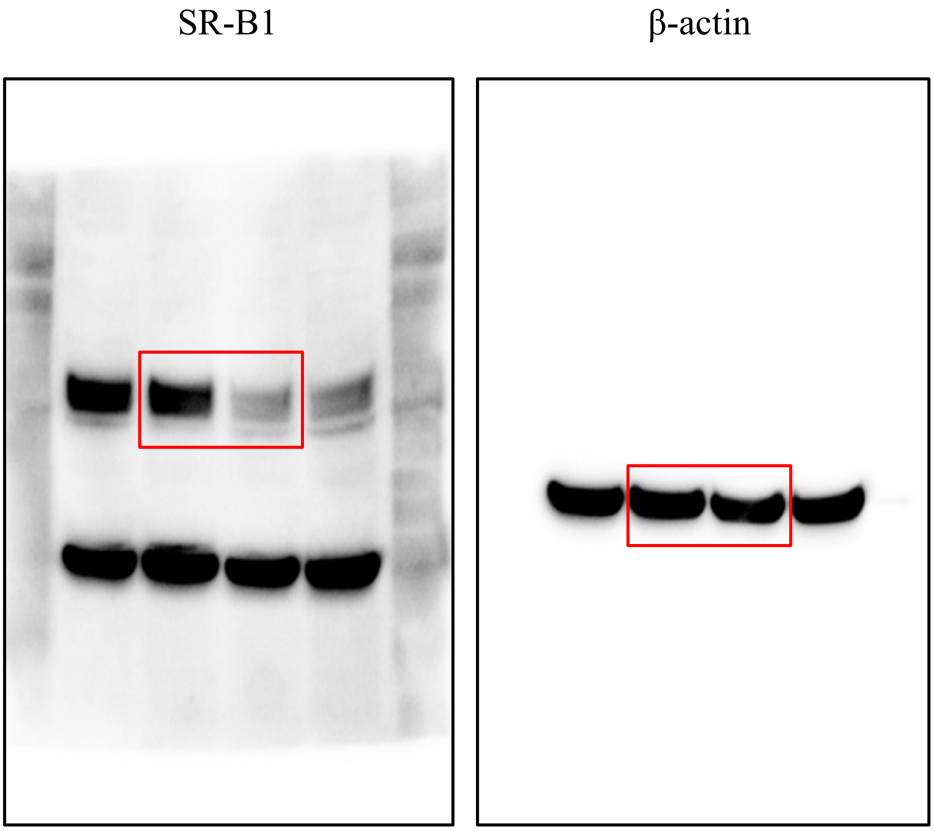

Supplement: Supplementary file 1 — Supplementary Information [file 41598_2018_38014_MOESM1_ESM.pdf]
